# Iatrogenic Cushing’s Syndrome and the Hidden Ingredient of Artri King

**DOI:** 10.7759/cureus.100843

**Published:** 2026-01-05

**Authors:** Basma Ataallah, Mohammed Al Tameemi

**Affiliations:** 1 Endocrinology, Diabetes and Metabolism, Houston Methodist Hospital, Houston, USA; 2 Endocrinology, Diabetes and Metabolism, Kelsey-Seybold Clinic, Houston, USA

**Keywords:** adrenal suppression, artri king, dietary supplements, exogenous glucocorticoids, fragility fractures, hidden steroids, hypercortisolism, hypothalamic–pituitary–adrenal axis, iatrogenic cushing’s syndrome, osteoporosis

## Abstract

Cushing’s syndrome is a rare disorder caused by prolonged exposure to glucocorticoids, either from endogenous overproduction or exogenous sources, with exogenous steroid use being the most common etiology. Clinical manifestations may include moon facies, abdominal striae, easy bruising, muscle weakness, and complications such as osteoporosis and fragility fractures. Many remedies and supplements marketed for inflammatory conditions are sold online or over the counter, and some may contain hidden or undisclosed steroids that can lead to hypercortisolism. We present a case of a 52-year-old man with osteoporosis who sustained fragility fractures and became wheelchair-bound due to progressive lower extremity weakness. Evaluation demonstrated suppression of the hypothalamic-pituitary-adrenal axis, with undetectable salivary and urinary cortisol levels. Further investigation revealed long-term use of Artri King, a supplement for musculoskeletal pain that contains undisclosed glucocorticoids. This case highlights the risk of unregulated supplements causing iatrogenic Cushing’s syndrome and its associated complications.

## Introduction

Cushing’s syndrome represents a constellation of signs and symptoms resulting from prolonged exposure to glucocorticoids [1]. Common manifestations may include moon facies, facial plethora, abdominal striae, easy bruising, and proximal muscle weakness [1]. Etiologies may be adrenocorticotropic hormone (ACTH)-dependent, originating from pituitary or ectopic sources, or ACTH-independent, such as adrenal pathology. In everyday clinical practice, however, exogenous glucocorticoid exposure remains the most common cause [2,3].

Exogenous steroids are available in multiple formulations, including oral, parenteral, inhaled, and topical preparations, and may be prescribed by healthcare providers or found in commercial products sold online or over the counter [4]. Prolonged exposure can result in hypercortisolism and its associated complications [5]. Therefore, careful assessment for exogenous steroid use is essential when evaluating patients with suspected Cushing’s syndrome. We report a case of iatrogenic Cushing’s syndrome secondary to the use of Artri King, a “herbal” supplement containing undisclosed glucocorticoids.

## Case presentation

A 52-year-old male with a history of prediabetes presented with osteoporosis and fragility fractures. Osteoporosis was diagnosed during imaging performed for the evaluation of back pain, which revealed thoracic spine compression fractures as well as a healed rib fracture. As a result, he became wheelchair-bound due to progressive lower extremity weakness. The patient denied prior trauma and had no family history of osteoporosis or pathologic fractures. He denied the use of steroids, proton pump inhibitors, anticoagulants, or antiseizure medications. He did not smoke and reported no alcohol use. There was no history of hypogonadism, bone disease, or fractures during childhood. Biochemical evaluation revealed a normal complete blood count, with pertinent laboratory results summarized in Table 1.

**Table 1:** Biochemical laboratory results. *: Reference intervals may vary by assay method and laboratory.

Laboratory test	Value	Units	Reference range
Total testosterone	415	ng/dL	264–916
Intact parathyroid hormone	9.4	pg/mL	8.7–77.1
Corrected serum calcium	9.6	mg/dL	8.6–10.3
24-hour urine calcium	144	mg/24 hours	100–300*
Plasma adrenocorticotropic hormone	Undetectable	pg/mL	7–63*
Late-night salivary cortisol	Undetectable	µg/dL	≤0.09*
24-hour urine free cortisol	Undetectable	µg/24 hours	10–50*

**Figure 1:**
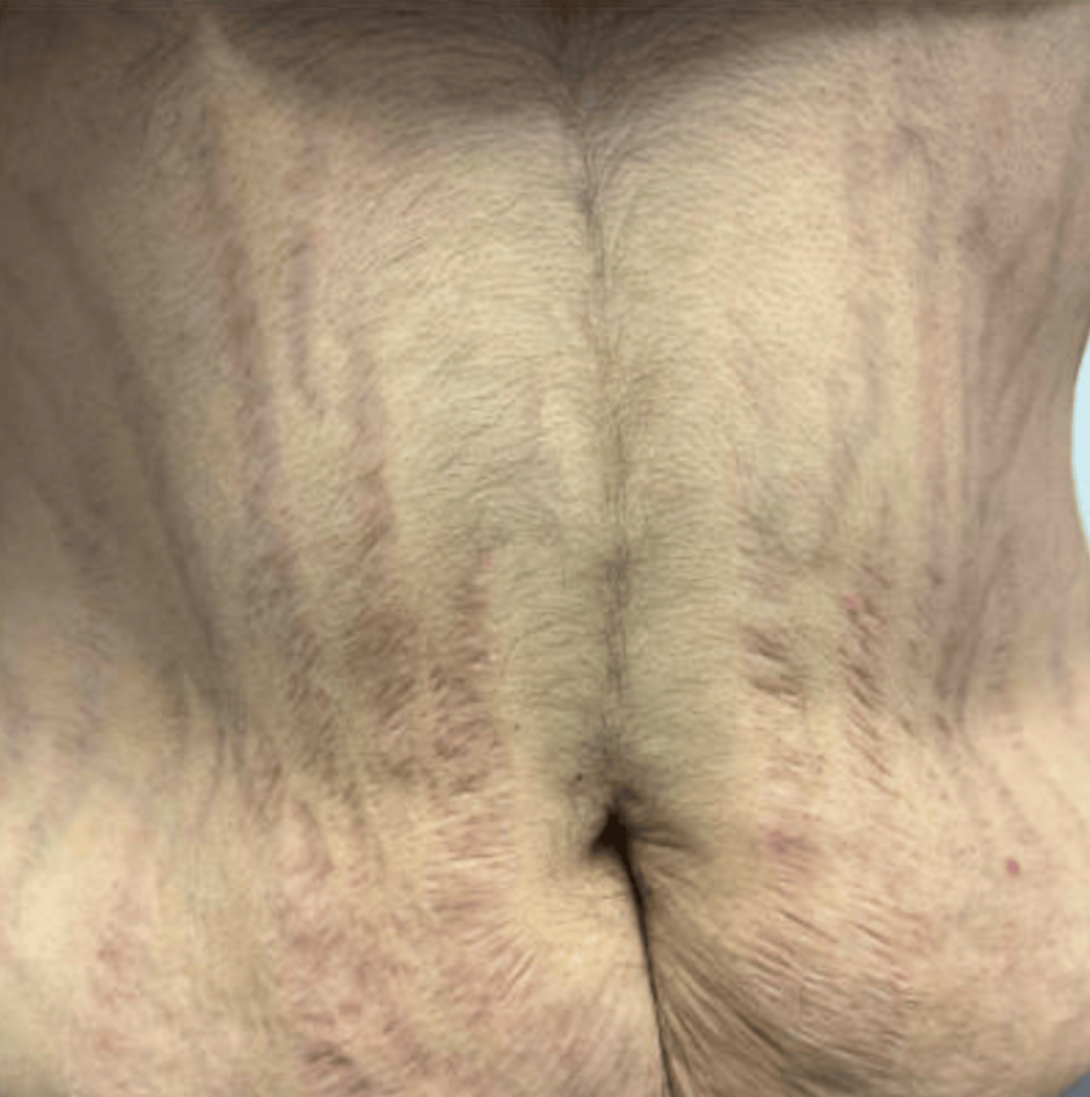
Purplish (violaceous) abdominal striae over the abdomen.

Given the presence of fragility fractures and physical examination findings consistent with Cushing’s syndrome, including moon facies, dorsocervical and supraclavicular fat fullness, and purplish striae (Figure 1), further evaluation was pursued. Laboratory testing demonstrated an undetectable serum ACTH level, and both late-night salivary cortisol and 24-hour urinary free cortisol levels were undetectable, raising concern for exogenous glucocorticoid exposure (Table 1). Dual-energy X-ray absorptiometry demonstrated a spinal bone mineral density of 0.686 g/cm² with a T-score of −3.7.

On further questioning, the patient reported taking Artri King for two years, obtained from Mexico, for joint pain and arthritis. A review of U.S. Food and Drug Administration (FDA) reports confirmed that Artri King contains hidden ingredients, including dexamethasone, not listed on its label. The supplement was discontinued, and the patient was started on a gradual steroid taper to minimize glucocorticoid withdrawal symptoms and allow for the recovery of hypothalamic-pituitary-adrenal (HPA) axis function.

## Discussion

Cushing’s syndrome is a rare disorder characterized by a constellation of signs and symptoms affecting multiple organ systems as a result of prolonged exposure to excess cortisol. Hypercortisolism may result from endogenous overproduction of cortisol or from exposure to exogenous glucocorticoids [1]. Regardless of etiology, clinical manifestations commonly include moon facies, abdominal striae, truncal obesity, and easy bruising [1]. Patients with Cushing’s syndrome may also develop complications such as hyperglycemia, uncontrolled hypertension, proximal muscle weakness, and reduced BMD, which can lead to fragility fractures [2]. These complications significantly impair quality of life and may be fatal if the condition is not diagnosed and treated promptly [3].

Endogenous hypercortisolism is less common, with an estimated incidence of 2-3 cases per million per year [4]. However, recent studies suggest a higher prevalence among individuals with diabetes mellitus, osteoporosis, particularly those with fragility fractures, and hypertension [5]. Cushing’s syndrome can be classified as ACTH-dependent, in which ACTH originates from the pituitary gland or an ectopic source, or ACTH-independent, typically due to adrenal adenoma, adrenal hyperplasia, or adrenal carcinoma [5]. Although exogenous glucocorticoid exposure is the most common cause of Cushing’s syndrome, the true incidence of iatrogenic Cushing’s syndrome remains unknown [6]. Rarely, Cushing’s syndrome may result from concurrent exogenous steroid use and endogenous cortisol overproduction, which presents diagnostic challenges [6].

Glucocorticoid-containing medications are widely used in the management of inflammatory diseases, malignancies, and post-transplant care [7,8]. All forms of exogenous glucocorticoids, including oral, inhaled, injectable, and topical preparations, can cause features of hypercortisolism when used at high doses or for prolonged periods [9-12]. Extended exposure, particularly at higher doses, may also result in secondary adrenal insufficiency, even with topical formulations [13]. In addition to conventional glucocorticoids, other medications may induce iatrogenic hypercortisolism; for example, high-dose megestrol exhibits glucocorticoid-like activity and can produce Cushing’s syndrome-like features [14]. Furthermore, drugs that inhibit cytochrome P450 metabolism, such as itraconazole, can impair steroid clearance and increase systemic glucocorticoid exposure [15].

Of increasing concern is the availability of steroid-containing supplements sold over the counter or online without prescription [16]. These products are commonly marketed for conditions such as arthritis and other inflammatory disorders [16]. Prolonged use may cause Cushing’s syndrome with complications such as skin atrophy, obesity, myopathy, and fractures. The U.S. FDA has issued multiple warnings regarding dietary supplements and conventional foods found to contain undisclosed pharmaceutical ingredients [17]. A 2016 study evaluating 12 over-the-counter “adrenal support” supplements in the United States found that most contained at least one steroid hormone [18]. Another analysis of FDA warnings on unapproved pharmaceutical ingredients reported that 37.5% of products marketed for inflammatory conditions, including joint and muscle pain, contained dexamethasone [19]. Among these products, Artri King, marketed for joint pain and arthritis, has been associated with multiple FDA reports of adverse events due to undisclosed dexamethasone and methylprednisolone. These supplements remain widely available online, in select retail stores, and internationally [20].

## Conclusions

This case highlights the importance of considering unregulated supplements as a potential source of exogenous glucocorticoids in patients presenting with osteoporosis and unexplained fragility fractures. Although the patient initially denied steroid use, detailed history revealed prolonged exposure to Artri King, resulting in iatrogenic Cushing’s syndrome with HPA axis suppression. Before discontinuation of steroid-containing supplements, evaluation for adrenal insufficiency is essential. Gradual tapering of glucocorticoids remains the standard approach to prevent withdrawal symptoms and support recovery of adrenal function.

## References

[1] Nieman LK (2018). Recent updates on the diagnosis and management of Cushing's syndrome. Endocrinol Metab (Seoul).

[2] Dunn C, Amaya J, Green P (2023). A case of iatrogenic Cushing's syndrome following use of an over-the-counter arthritis supplement. Case Rep Endocrinol.

[3] Castinetti F, Morange I, Conte-Devolx B, Brue T (2012). Cushing's disease. Orphanet J Rare Dis.

[4] Nieman LK, Biller BM, Findling JW, Newell-Price J, Savage MO, Stewart PM, Montori VM (2008). The diagnosis of Cushing's syndrome: an Endocrine Society Clinical Practice Guideline. J Clin Endocrinol Metab.

[5] Manubolu S, Nwosu O (2017). Exogenous Cushing’s syndrome secondary to intermittent high dose oral prednisone for presumed asthma exacerbations in the setting of multiple emergency department visits. J Clin Transl Endocrinol Case Rep.

[6] Tong CV, Rajoo S (2019). Co-occurrence of exogenous and endogenous Cushing's syndromes-dilemma in diagnosis. Case Rep Endocrinol.

[7] Broersen LH, Pereira AM, Jørgensen JO, Dekkers OM (2015). Adrenal insufficiency in corticosteroids use: systematic review and meta-analysis. J Clin Endocrinol Metab.

[8] Yasir M, Goyal A, Sonthalia S (2025). Corticosteroid Adverse Effects. https://www.ncbi.nlm.nih.gov/books/NBK531462/.

[9] Dow A, Yu R, Carmichael J (2013). Too little or too much corticosteroid? Coexisting adrenal insufficiency and Cushing's syndrome from chronic, intermittent use of intranasal betamethasone. Endocrinol Diabetes Metab Case Rep.

[10] Hopkins RL, Leinung MC (2005). Exogenous Cushing's syndrome and glucocorticoid withdrawal. Endocrinol Metab Clin North Am.

[11] Hughes JM, Hichens M, Booze GW, Thorner MO (1986). Cushing's syndrome from the therapeutic use of intramuscular dexamethasone acetate. Arch Intern Med.

[12] Weber SL (1997). Cushing'S syndrome attributable to topical use of lotrisone. Endocr Pract.

[13] Pektas SD, Dogan G, Cinar N (2017). Iatrogenic Cushing's syndrome with subsequent adrenal insufficiency in a patient with psoriasis vulgaris using topical steroids. Case Rep Endocrinol.

[14] Steer KA, Kurtz AB, Honour JW (1995). Megestrol-induced Cushing's syndrome. Clin Endocrinol (Oxf).

[15] Bolland MJ, Bagg W, Thomas MG, Lucas JA, Ticehurst R, Black PN (2004). Cushing's syndrome due to interaction between inhaled corticosteroids and itraconazole. Ann Pharmacother.

[16] Saad-Omer SM, Kinaan M, Matos M, Yau H (2023). Exogenous Cushing syndrome and hip fracture due to over-the-counter supplement (Artri King). Cureus.

[17] Patel R, Sherf S, Lai NB, Yu R (2022). Exogenous Cushing syndrome caused by a "herbal" supplement. AACE Clin Case Rep.

[18] Akturk HK, Chindris AM, Hines JM, Singh RJ, Bernet VJ (2018). Over-the-counter "adrenal support" supplements contain thyroid and steroid-based adrenal hormones. Mayo Clin Proc.

[19] Tucker J, Fischer T, Upjohn L, Mazzera D, Kumar M (2018). Unapproved pharmaceutical ingredients included in dietary supplements associated with US Food and Drug Administration warnings. JAMA Netw Open.

[20] (2025). U.S. Food and Drug Administration. Public Notification: Artri King contains hidden drug ingredients. https://www.fda.gov/drugs/medication-health-fraud/public-notification-artri-king-contains-hidden-drug-ingredients.

